# Availability and reporting quality of external validations of machine-learning prediction models with orthopedic surgical outcomes: a systematic review

**DOI:** 10.1080/17453674.2021.1910448

**Published:** 2021-04-18

**Authors:** Olivier Q Groot, Bas J J Bindels, Paul T Ogink, Neal D Kapoor, Peter K Twining, Austin K Collins, Michiel E R Bongers, Amanda Lans, Jacobien H F Oosterhoff, Aditya V Karhade, Jorrit-Jan Verlaan, Joseph H Schwab

**Affiliations:** a Orthopedic Oncology Service, Massachusetts General Hospital, Harvard Medical School, Boston, USA;;; b Department of Orthopedic Surgery, University Medical Center Utrecht, Utrecht University, The Netherlands

## Abstract

Background and purpose — External validation of machine learning (ML) prediction models is an essential step before clinical application. We assessed the proportion, performance, and transparent reporting of externally validated ML prediction models in orthopedic surgery, using the Transparent Reporting for Individual Prognosis or Diagnosis (TRIPOD) guidelines.

Material and methods — We performed a systematic search using synonyms for every orthopedic specialty, ML, and external validation. The proportion was determined by using 59 ML prediction models with only internal validation in orthopedic surgical outcome published up until June 18, 2020, previously identified by our group. Model performance was evaluated using discrimination, calibration, and decision-curve analysis. The TRIPOD guidelines assessed transparent reporting.

Results — We included 18 studies externally validating 10 different ML prediction models of the 59 available ML models after screening 4,682 studies. All external validations identified in this review retained good discrimination. Other key performance measures were provided in only 3 studies, rendering overall performance evaluation difficult. The overall median TRIPOD completeness was 61% (IQR 43–89), with 6 items being reported in less than 4/18 of the studies.

Interpretation — Most current predictive ML models are not externally validated. The 18 available external validation studies were characterized by incomplete reporting of performance measures, limiting a transparent examination of model performance. Further prospective studies are needed to validate or refute the myriad of predictive ML models in orthopedics while adhering to existing guidelines. This ensures clinicians can take full advantage of validated and clinically implementable ML decision tools.

Multiple machine learning (ML) algorithms have recently been developed for prediction of outcomes in orthopedic surgery. A recent systematic review demonstrated that 59 models are currently available covering a wide variety of surgical outcomes, such as survival, postoperative complications, hospitalization, or discharge disposition to aid clinical decision-making (Ogink et al. 2021). However, it is imperative that these models are accurate, reliable, and applicable to patients outside the developmental dataset. Even though internal validation studies regularly report good performance, these results are often too optimistic as performance on external validation worsens due to initial overfitting (Collins et al. 2014, Siontis et al. 2015).

External validation refers to assessing the model’s performance on a dataset that was not used during development. Testing the developed model on independent datasets addresses the aforementioned concerns of internal validation, including: the generalizability of the model in different patient populations, shortcomings in statistical modelling (e.g., incorrect handling of missing data), and model overfitting (Collins et al. 2014, 2015). Therefore, external validation is essential before a model can be used in routine clinical practice.

Although a growing number of ML prediction models are being developed in orthopedics, no overview exists of the number of available ML prediction models that are externally validated, how they perform in an independent dataset, and what the transparency of reporting is of these external validation studies. Therefore, we assessed the proportion, performance, and transparent reporting of externally validated ML prediction models in orthopedic surgery, using the Transparent Reporting of a multivariable prediction model for Individual Prognosis or Diagnosis (TRIPOD) guidelines.

## Material and methods

### Systematic literature search

Adhering to the 2009 PRISMA guidelines, this review was registered online at PROSPERO (Moher et al. 2016). A systematic search was conducted in PubMed, Embase and Cochrane up to November 17, 2020.

**Figure 1.**
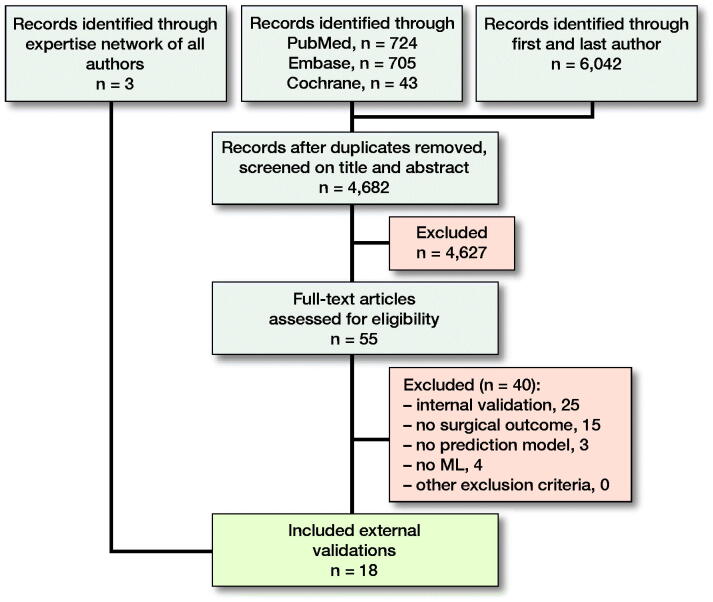
Flowchart of study selection.

3 different domains of medical subject headings (MeSH) terms and keywords were combined with “AND”, and within domains the terms were combined with ‘OR’. The 3 domains included words related to orthopedics, ML, and external validation. In addition, we searched the first and last authors from the 59 ML prediction models previously identified in a systematic review by our study group combined with the domain “machine learning” (Appendix 1, see Supplementary data) (Ogink et al. 2021). 2 authors (NDK, PKT) independently screened all titles and abstracts. All references of the included studies were examined for relevant studies not identified by the initial search. The final list of included studies was sent to all coauthors, all of whom had worked with and/or published ML prediction models in orthopedics for a last check of potentially missed studies (Figure 1).

### Eligibility criteria

Inclusion criteria were: external validation; prediction models based on ML; and orthopedic surgical outcome (defined as any outcome after musculoskeletal surgery). Exclusion criteria were: non-ML prediction model (e.g., standard logistic regression); internal validation (e.g., cross-validation and holdout test set from developmental dataset); lack of full text; conference abstracts; animal studies; and languages other than English, Spanish, German, or Dutch. We considered advanced logistic regression methods as ML algorithms such as penalized LR (LASSO, ridge or elastic-net), boosted LR and bagged LR.

### Data extraction

Data extracted from each study were: year of publication; 1st author; disease; type of surgery; prospective study design; level of care from which the dataset originates (e.g., tertiary); country; type of ML algorithm (e.g., Bayesian Belief Network); sample size; input features; predicted outcome; time points of outcome; performance measures according to the ABCD approach (Steyerberg and Vergouwe 2014) (A = calibration-in-the-large, or the model intercept; B = calibration slope; C = discrimination, with an area under the curve [AUC] using evaluation metrics of receiver operating characteristic [ROC] curves or precision-recall [PR] plots; D = decision-curve analysis); mention of guideline adherence; TRIPOD items (Collins et al. 2015); and PROBAST domains (Wolff et al. 2019). Data were extracted from the largest cohort when multiple cohorts were present and the best performing model if a study reported results for multiple outcomes (e.g., 90-day and 1-year survival). Performance measures of the developmental study were extracted to compare with the results of external validation. 2 reviewers (OQG, BJJB) independently extracted all data and disagreements were discussed with a third reviewer present (PTO) until consensus was achieved.

### TRIPOD and PROBAST

The TRIPOD guidelines were simultaneously published in 11 leading medical journals in January 2015 (Collins et al. 2015). Although various other guidelines exist (von Elm et al. 2007, Luo et al. 2016), we deemed the TRIPOD guidelines essential for transparent reporting requirements, which is imperative when judging the validity and applicability of a prediction model. Also, the TRIPOD guidelines were developed entirely for transparent reporting of prognosis or diagnosis prediction model studies (Figure 2 and 3, see Supplementary data).

The PROBAST assesses the risk of bias of a study that validates a prognostic prediction model (Wolff et al. 2019). It is specifically designed to grade studies included in a systematic review. 4 domains are assessed for risk of bias: (1) participants; (2) predictors; (3) outcome; (4) and analysis (Figure 4, see Supplementary data).

### Statistics

The proportion of externally validated ML prediction models in orthopedic surgical outcome was calculated by dividing 59 models by the externally validated models identified through this current study. Our group previously found 59 ML prediction models using only internal validation meeting the same criteria (except the criterium was “developmental” instead of “external validation”) in a systematic search dated up until June 18, 2020 (Groot et al. 2021, Ogink et al. 2021). Of the identified external validation studies, we determined how many unique models were externally validated, as 1 model can be externally validated multiple times with different datasets. 1 incremental value study was found, which also reported on external validation. Only the external validation part was assessed.

Performance measures were extracted and expressed as they were originally reported (Steyerberg and Vergouwe 2014). No meta-analysis could be performed because of obvious heterogeneity between studies. Adherence to the TRIPOD guidelines and PROBAST domains was expressed in percentages and visualized by graphs.

We used Microsoft Excel Version 19.11 (Microsoft Corp, Redmond, WA, USA) to extract data using standardized forms, and to create all figures and tables, and Mendeley Desktop Version 1.19.4 (Mendeley, London, UK) as reference software.

### Ethics, funding, and potential conflicts of interest

As there was no contact with patients and no study interventions were performed, permission from our institutional review board was not required. The study was supported by a grant from the Foundation “De Drie Lichten” in The Netherlands (€7.195). The authors reported no further funding disclosures or conflicts of interest.

## Results

### Study characteristics

**Figure 6.**
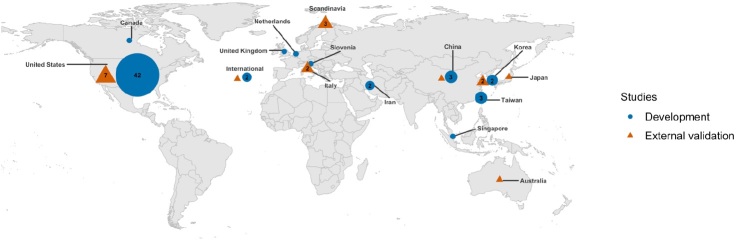
Distribution of development and external validation studies. All of the developmental studies that were externally validated except 2 South Korean ones were built on American datasets, unlike the origin of the external validation studies. Symbols without a number correspond with 1 study. Studies that included both development and external validation within the same study were counted twice in the figure according to where both datasets originated from.

4,682 unique studies were identified of which 15 remained after full-text screening. 3 studies missed by the search were added by the coauthor’s expertise network (Forsberg et al. 2012, 2017, Piccioli et al. 2015, Ogura et al. 2017, Bongers et al. 2019, Harris et al. 2019, Huang et al. 2019, Jo et al. 2019, Meares et al. 2019, Ramkumar et al. 2019a, 2019b, Stopa et al. 2019, Anderson et al. 2020, Bongers et al. 2020a, 2020b, Ko et al. 2020, Karhade et al. 2020, Overmann et al. 2020). None of the external validations used a prospective cohort and 12/18 investigated survival in bone oncology (Table 1). 6/18 mentioned adherence to the TRIPOD guidelines, but none included the actual checklist. All studies were affiliated with 6 institutions of which 7/18 with PATHFx and 5/18 with SORG (Figure 5, see Supplementary data). 17/18 had at least 1 author who was also an author on the paper that developed the model being evaluated. 9/18 of the studies reported on both development and external validation in the same paper; the other 9 only reported on external validation. All of the ML prediction models were freely available at www.pathfx.org, www.sorg-ai.com, safetka.net/, http://med.stanford.edu/s-spire/Resources/clinical-tools-.html, and https://github.com/JaretK/NeuralNetArthroplasty. 17 datasets were used because 3 studies used 1 Scandinavian dataset and 1 study included 2 validation registry cohorts (Table 2). 14/17 of the datasets originated from hospitals, the other 3 were from a registry. The median sample size of the external validation datasets was 274 patients (IQR, 178–552) and 7/17 were American datasets (Figure 6).

**Table 1.** Characteristics of external validation studies on orthopedic surgical outcome prediction (n = 18)

First author publication year,	Disease condition	Operation	ML model	Prospective database	Output	Input predictors	Number of patients	Adherence to aguideline
Anderson, 2020	Pathological fractures	nos	BBN	no	Survival	Clinical	197	TRIPOD
Bongers, 2019	Extracranial chondrosarcoma	nos	BPM	no	Survival	Clinical	179	none
Bongers, 2020a	Extracranial chondrosarcoma	nos	BPM	no	Survival	Clinical	464	TRIPOD
Bongers, 2020b	Bone metastases (spine)	nos	SGB	no	Survival	Clinical	200	TRIPOD
Forsberg, 2012	Bone metastases (extremities)	nos	BBN	no	Survival	Clinical	815	none
Forsberg, 2017	Bone metastases	nos	BBN	no	Survival	Clinical	815	TRIPOD
Harris, 2019	nos	Elective TJA	LASSO	no	Survival, complications	Clinical	70,569	none
Huang, 2019	Non-metastatic chondrosarcoma	nos	LASSO	no	Survival	Clinical, surgical	72	none
Jo, 2020	nos	TKA	GBM	no	Transfusion	Clinical, surgical	400	none
Karhade, 2020	Bone metastases (spine)	nos	SGB	no	Survival	Clinical	176	TRIPOD
Ko, 2020	nos	TKA	GBM	no	Acute kidney injury	Clinical, surgical	455	none
Meares, 2019	Bone metastases (femoral)	nos	BBN	no	Survival	Clinical	114	none
Ogura, 2017	Bone metastases	nos	BBN	no	Survival	Clinical	261	none
Overmann, 2020	Bone metastases (extremities)	nos	BBN	no	Survival	Clinical	815	none
Piccioli, 2015	Bone metastases	nos	BBN	no	Survival	Clinical	287	none
Ramkumar, 2019a	Osteoarthritis	THA	ANN	no	LOS; discharge disposition	Clinical	2,771	none
Ramkumar, 2019b	Osteoarthritis	TKA	ANN	no	LOS; discharge disposition	Clinical	4,017	none
Stopa, 2019	Lumbar disc disorder	Decompression or fusion	NN	no	Nonhome discharge	Clinical, surgical	144	TRIPOD

ML = machine learning; nos = not otherwise specified; TJA = total joint arthroplasty; TKA = total knee arthroplasty; THA = total hip arthroplasty; BBN = Bayesian Belief Network; NN = neural network; BPM = Bayes Point Machine; SGB = Stochastic Gradient Boosting; LASSO = least absolute shrinkage and selection operator; GBM = gradient boosting machine; LOS = length of stay; TRIPOD = Transparent Reporting of a multivariable prediction model for Individual Prognosis Or Diagnosis.

**Table 2.** Characteristics of hospital setting and years of enrollment from external validation and corresponding developmental studies

Model or institution		Authors’ development and validation the same	First author, publication	Country	Tertiary	Hospitals	Registry	Years of year enrollment
Cleveland	Validation	yes	Ramkumar, 2019a	USA	mixed	11	no	2016–2018
	Development		Same	USA	mixed	multiple	NIS	2009–2011
Cleveland	Validation	yes	Ramkumar, 2019b	USA	mixed	11	no	2016–2018
	Development		Same	USA	mixed	multiple	NIS	2009–2013
BETS/PATHFx 1.0	Validation	yes	Forsberg, 2012	Scandinavia	yes	8	no	1999–2009
	Development		Forsberg, 2011	USA	yes	1	no	1999–2003
PATHFx 1.0	Validation	yes	Piccioli, 2015	Italy	yes	13	no	2010–2013
	Development		Forsberg, 2011	USA	yes	1	no	1999–2003
PATHFx 1.0	Validation	yes	Forsberg, 2017	Scandinavia	yes	8	no	1999–2009
	Development		Same	USA	yes	1	no	1999–2003
PATHFx 1.0	Validation	yes	Ogura, 2017	Japan	yes	5	no	2009–2015
	Validation	no	Meares, 2019	Australia	unknown	1	no	2003–2014
	Development		Forsberg, 2011/2017	USA	yes	1	no	1999–2003
PATHFx 2.0	Validation	yes	Overmann, 2020	Scandinavia	yes	8	no	1999–2009
	Development		Same	USA	yes	1	no	1999-2003
PATHFx 3.0	Validation	yes	Anderson, 2020	Multinational	yes	multiple	IBMR^a^	2016–2018
	Development		Same	USA	yes	1	no	1999–2003,
								2015–2018
SafeTKA	Validation	yes	Jo, 2020	unknown	unknown	1	no	unknown
	Development		Same	South-Korea	yes	1	no	2012–2018
SafeTKA	Validation	yes	Ko, 2020	South-Korea	yes	1	no	2018–2019
	Development		Same	South-Korea	yes	2	no	2012–2019
SORG	Validation	yes	Bongers, 2019	USA	yes	2	no	1992–2013
	Development		Thio, 2018	USA	mixed	multiple	SEER	2000–2010
SORG	Validation	yes	Bongers, 2020a	Italy	yes	1	no	2000–2014
	Validation	yes	Karhade, 2020	USA	yes	1	no	2003–2016
	Development		Karhade, 2020	USA	yes	2	no	2000–2016
SORG	Validation	yes	Bongers, 2020b	USA	yes	1	no	2014–2016
	Validation	yes	Stopa, 2019	USA	yes	1	no	2013–2015
	Development		Karhade, 2018	USA	mixed	multiple	NSQIP	2011–2016
Stanford	Validation	yes	Harris, 2019	USA	mixed	multiple	VASQIP	2005–2013
	Development		Same	USA	mixed	multiple	NSQIP	2013–2014
Zhengzhou	Validation	yes	Huang, 2019	China	yes	1	no	2011–2016
	Development		Same	USA	mixed	multiple	SEER	2005–2014

BETS = Bayesian Estimated Tools for Survival; SORG = Spinal Oncology Research Group; NSQIP = National Surgical Quality Improvement Program; SEER = Surveillance, Epidemiology, and End Results; IBMR = International Bone Metastasis Registry; NIS = National Inpatient Sample; VASQIP = Veterans Affairs Surgical Quality Improvement Program.

^a^This study also included an external validation on a second registry cohort of 192 patients from the Military Health System Data Repository.

### Proportion

This systematic review identified 18 external validation studies of ML models predicting outcomes in orthopedic surgery. In these 18 external validation studies, 10 unique ML prediction models were validated as 2 models were validated twice, and 1 model 7 times as it was validated and updated multiple times with distinct datasets. Therefore, 10/59 of the ML models predicting outcomes in orthopedic surgery published until June 18, 2020 were externally validated. Of the 10 models, 3 were externally validated with patients from another country than the developmental cohort, including 1 model by 4 different countries.

### Performance

All studies reported the ROC AUC, which retained good discriminative ability with a value greater than 0.70 and/or less than 0.10 decreased performance compared with the corresponding development study (Table 3 and Figure 7, see Supplementary data). No PR AUC evaluation metrics were provided, despite 3/18 of the datasets consisting of imbalanced class distribution in which the ratio events:non-events was greater than 1:10. Calibration intercept and slope, or curve, were reported in 7/18. 5/18 reported calibration slope or curves that showed overall underfitting of the data. Decision curve analyses were provided in 9/18, all of which illustrated that the prediction models were suitable for clinical use.

### TRIPOD and PROBAST

**Figure 8.**
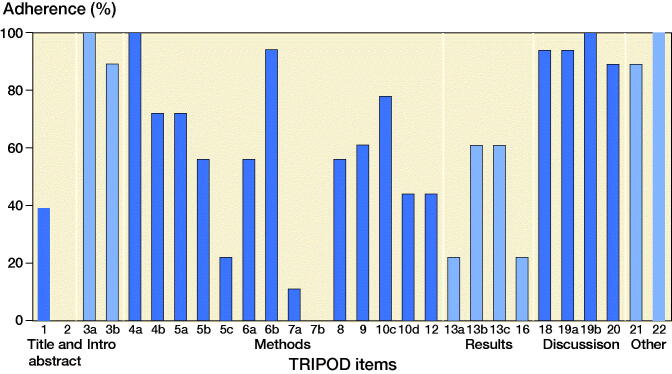
Overall adherence to each TRIPOD item (n = 18).

The overall median completeness of the TRIPOD items was 61% (IQR 43–90%; Figure 8 and Table 4, see Supplementary data). All method items adhered to a median completeness of 56% (IQR 44–72%) and all results items to a median of 42% (IQR 22–61%). 6 items were reported in more than 16 studies including 3 discussion items (Table 5). 6 items were reported in less than 4 studies, including details of abstract, participant selection, and reporting key performance measures.

**Table 5.** Sorted by completeness of above 90% reporting and under 25% of individual TRIPOD items

TRIPOD item	TRIPOD description	% (n)
**Complete reporting > 90%**		
3a	Explain the medical context (including whether diagnostic or prognostic) and rationale for validating the multivariable prediction model, including references to existing models	100 (18)
4a	Describe the study design or source of data (e.g., randomized trial, cohort, or registry data), separately for the validation data set	100 (18)
19b	Give an overall interpretation of the results considering objectives, limitations, results from similar studies and other relevant evidence	100 (18)
22	Give the source of funding and the role of the funders for the present study	100 (18)
6b	Report any actions to blind assessment of the outcome to be predicted	94 (17)
19a	Discuss the results with reference to performance in the development data, and any other validation data	94 (17)
**Complete reporting < 25%**		
2	Provide a summary of objectives, study design, setting, participants, sample size, predictors, outcome, statistical analysis, results, and conclusions	0 (0)
7b	Report any actions to blind assessment of predictors for the outcome and other predictors	0 (0)
7a	Clearly define all predictors used in validating the multivariable prediction model, including how and when they were measured	11 (2)
5c	Give details of treatments received, if relevant	22 (4)
13a	Describe the flow of participants through the study, including the number of participants with and without the outcome and, if applicable, a summary of the follow-up time. A diagram may be helpful	22 (4)
16	Report performance measures (with confidence intervals) for the prediction model (results)	22 (4)

TRIPOD = Transparent Reporting of a multivariable prediction model for Individual Prognosis Or Diagnosis.

**Figure 9.**
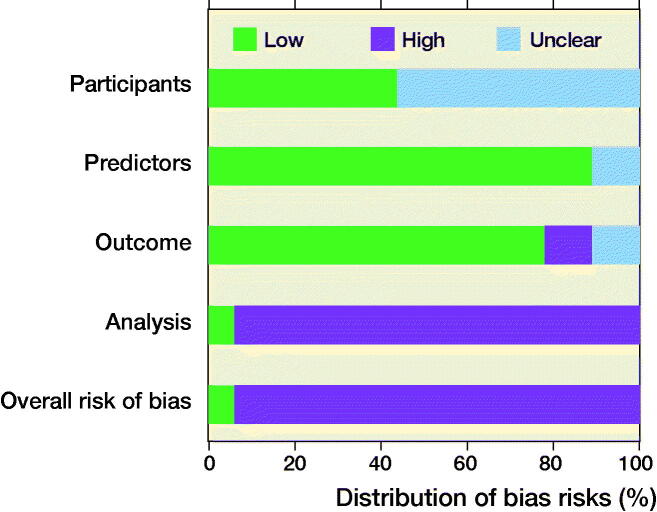
PROBAST results for all 4 domains and overall judgement (n = 18).

Participant selection (domain 1) was considered an unclear risk of bias in 10 studies because no information was provided on the inclusion and exclusion of patients (Figure 9). Predictors (domain 2) were deemed a low risk of bias in 16 studies, as 2 studies were unclear in their predictor’s definitions and assessment. Outcome (domain 3) was rated a high risk of bias in 2 studies as they did not determine survival in a similar way for all patients by assigning “death” to all patients lost to follow-up. 2 additional studies in the outcome domain were rated an unclear risk of bias because it was difficult to discern if they used the same postoperative complication definitions for both the development and external validation study. Analysis (domain 4) was rated a high risk of bias in 17 studies, mainly due to small sample sizes with less than 100 events in the outcome group or no calibration metrics. The overall judgement of risk of bias for the 18 studies was high in 17 studies and low in 1 study, as only 1 study scored “low risk of bias” across all 4 domains.

## Discussion

The focus on developing and publishing ML prediction models has led to an increasing body of studies. Yet, it is of equal importance to externally validate these models, as the TRIPOD states in its guidelines: “external validation is an invaluable and crucial step in the introduction of a new prediction model before it should be considered for routine clinical practice.” Although the external validation studies identified in this review retained good discriminatory performance and overall adhered well to the TRIPOD guidelines, only 10/50 of the ML models predicting orthopedic surgical outcome published up to June 2020 have been externally validated. Skepticism of these non-externally validated models is necessary and an increased effort in externally validating existing models is required to realize the full potential of ML prediction models.

### Proportion

A disappointingly low 10/59 of the current available ML prediction models were externally validated in orthopedic surgical outcome with none of the datasets being prospective. Prospectively testing the performance of ML models under real-world circumstances is an essential step towards integrating these models into the clinical setting and evaluating the impact on healthcare (Collins et al. 2015). In addition, increased effort towards external validation on patient data from distinct geographic sites is needed, as the generalizability of models to other countries may be affected by differences in healthcare systems, predictor measurements, and treatment strategies (Steyerberg et al. 2013). Although the recent surge of ML models in orthopedics is exciting, it is critical that these models are tested with external, real-world, operational data in different geographical settings before the orthopedic community can fully embrace the models in clinical practice.

### Performance

The external validations identified in this review retained good discrimination. Other key characteristics recommended evaluating a model’s performance such as calibration, and whether decision-curve analysis was inadequately or not reported, as observed here and in similar reviews (Collins et al. 2011, 2014, Bouwmeester et al. 2012, Tangri et al. 2013). Calibration measures were provided in only 7 of the 18 studies, preventing a transparent examination of model performance across the range of predicted probabilities (Steyerberg and Vergouwe 2014). Lastly, and arguably more important than the other metrics, is clinical usefulness evaluated by decision-curve analysis (Vickers and Elkin 2006). All 9 of the 18 studies that reported a decision-curve analysis indicated that the models were suitable for clinical use. Importantly, these curves do not estimate the likelihood of the outcome, but rather illustrate when the model should and should not be used in certain clinical situations over a range of thresholds. Overall, only 3 studies provided all 4 key measures to evaluate performance reliably, despite a substantial body of methodological literature and published guidance emphasizing the importance of these performance measures (von Elm et al. 2007, Steyerberg and Vergouwe 2014, Collins et al. 2015, Luo et al. 2016). Clinical researchers should use proposed frameworks such as Steyerberg’s ABCD approach to systematically report the performance of a validated model to allow accurate evaluation (Steyerberg and Vergouwe 2014).

An additional interesting find is that 17 of the 18 studies were conducted by authors involved in the development of the model. Authors evaluating their own model might be overly optimistic, selectively report the results to their own advantage, and even defer publication if the performance is poor (Siontis et al. 2015). Although validating one’s model is an essential first step, ideally this should be done by researchers not affiliated with the developmental study.

### TRIPOD and PROBAST

Although the external validations fared better in overall TRIPOD adherence than their corresponding developmental studies, they too had numerous incomplete items. The abstract, for which complete reporting required information on 12 elements, was incomplete in all studies. Some basic key details such as defining predictor definitions, outcome, or treatment elements were poorly reported, despite not being specific to ML external validation studies. Specifying and reporting performance measures was poorly done in over half of the studies. Despite 6 TRIPOD items scoring less than 25% (5 were methods/results), 11 items scored over 75%, which included mainly introduction and discussion items. This difference in adherence across sections perhaps illustrates that the orthopedic community comprehends the rationale, promise, and limitations of ML prediction models, but proper knowledge of methodological standards to describe and evaluate external validations studies is lacking. Standardized reporting and adherence to peer-reviewed guidelines such as the TRIPOD guidelines will aid in the execution and reporting of external validation studies, resulting in validated ML prediction models that are reliable, accurate, and that add to surgical decision-making (Collins et al. 2015).

The PROBAST domains identified 2 major concerns in addition to the TRIPOD items. First, little attention was given to the flow of patient selection, as none of the studies included a flow diagram of included and excluded patients. Possibly, studies purposely did not include flow diagrams or selection criteria to maintain the generalizability of the model to patients outside the selection criteria, but studies should explicitly state this. Second, the sample sizes were often too small, as only 5 of the 17 validation datasets had more than 100 events in each outcome group. Previous studies have shown that calibration results are less reliable with datasets with less than 100 outcome events (Vergouwe et al. 2005). In most circumstances, it would have been difficult to reach this number as the disease conditions were primarily bone oncology related. To address the issue of inadequate number of outcomes, multi-institutional collaboration is needed to achieve effective sample sizes to allow reliable external validations.

### Limitations

1st, studies meeting the selection criteria may have been missed. However, we believe this was unlikely as we used 4 different search strategies. In addition, we believe that any missed studies would not have had a profound impact on the review’s message as the percentage of externally validated models was well below 20%. 2nd, 5 of the 18 included studies originated from the authors’ institution (SORG) and the reviewers may have been biased assessing them. To account for this potential bias, the 2nd reviewer (BJJB) was not affiliated with the institution, the PI was not present during the consensus meetings, and an online PROSPERO protocol was registered. 3rd, publication bias may have occurred as successful external validations may be published more often. The performance results presented in this review may therefore be too optimistic and the number of studies externally validated too pessimistic. Studies demonstrating poorer performing models are part of the implementation process and ideally should be equally embraced by journals as high-performing models. In addition, the AUCs presented in 3 studies may have been too optimistic as they used ROC metrics on imbalanced datasets. Future studies should provide PR AUC metrics for datasets with an imbalanced class distribution (Saito and Rehmsmeier 2015). 4th, the presented low percentage of ML prediction models externally validated may have been unfair, as 20 ML models have been developed and published in the last year and external validation studies are time consuming. However, excluding the studies published in the last year to correct for this delay still only yielded a disappointing 18/39 of ML prediction models that were externally validated. In addition, not all published ML models are for deployment, as we are still exploring the potentials of ML and therefore publications’ primary motivation may be exploring the space of ML. Instead of externally validating these models, online tests should be provided where users can assess themselves how the ML models behave in different settings and parameters. Unfortunately, over half of the ML development studies did not provide online calculators, algorithms, and/or open access (Ogink et al. 2021). Future ML studies should place more emphasis on providing easy-to-access means where outside users can themselves assess model performance and behavior. 5th, various reporting guidelines exist such as STROBE and JMIR Guidelines for Developing and Reporting Machine Learning Models in Biomedical Research (von Elm et al. 2007, Luo et al. 2016). However, we used the TRIPOD guidelines to assess the transparent reporting as this guideline was explicitly developed to cover the development and validation of prediction models for prognosis (Collins et al. 2015). To improve on these guidelines, the TRIPOD authors are currently developing a TRIPOD-AI version specifically for reporting of AI prediction models (Collins and Moons 2019). 6th, the guidelines are endorsed by 21 medical journals, of which only 1 is orthopedic (Journal of Orthopedic & Sports Physical Therapy). Since none of the studies were published in journals that officially endorsed the TRIPOD, it may be unfair to expect compliance with these guidelines. However, we believe that the TRIPOD guidelines present a high-quality benchmark for assessing transparent reporting, which is necessary for externally validating existing models and creating clinically implementable ML prediction models. Despite these limitations, our review provides valuable insights into the amount and transparent reporting of current ML external validations in orthopedic surgical outcome prediction.

### Conclusion

Despite the evident importance of evaluating the performance of prediction models on unseen datasets, this is rarely done as institutions are protective of sharing their data and journals prefer publishing development studies. In addition, algorithms that perform poorly on external validation may be subject to publication bias. The handful of available external validation studies overall adhered well to the TRIPOD guidelines, but certain items that are essential for transparent reporting were inadequately reported or not reported at all, namely details of the abstract, participant selection, and key performance measures. Increased effort to externally validate existing models on large, prospective, geographically distinct datasets is required to ensure accurate and reliable validated ML prediction models. It will be difficult to achieve these types of datasets without multi-institutional collaboration across different geographic regions. We encourage researchers and institutions, from both within and outside the orthopedic ML community, to collaborate.

## Supplementary Material

Supplemental Material

## Supplementary data

Figures 2–5 and 7, Tables 3 and 4 and Appendix with search syntaxes are available as supplementary data in the online version of this article, http://dx.doi.org/10.1080/17453674.2021.1910448
