# Supplementary material for: Availability and reporting quality of external validations of machine-learning prediction models with orthopedic surgical outcomes: a systematic review
Source: Acta Orthop. 2021 Apr 18;92(4):385–93. doi: 10.1080/17453674.2021.1910448 (PMC8436968; doi:10.1080/17453674.2021.1910448)
Supplement: Supplemental Material [file IORT_A_1910448_SM7050.pdf]

Supplementary data

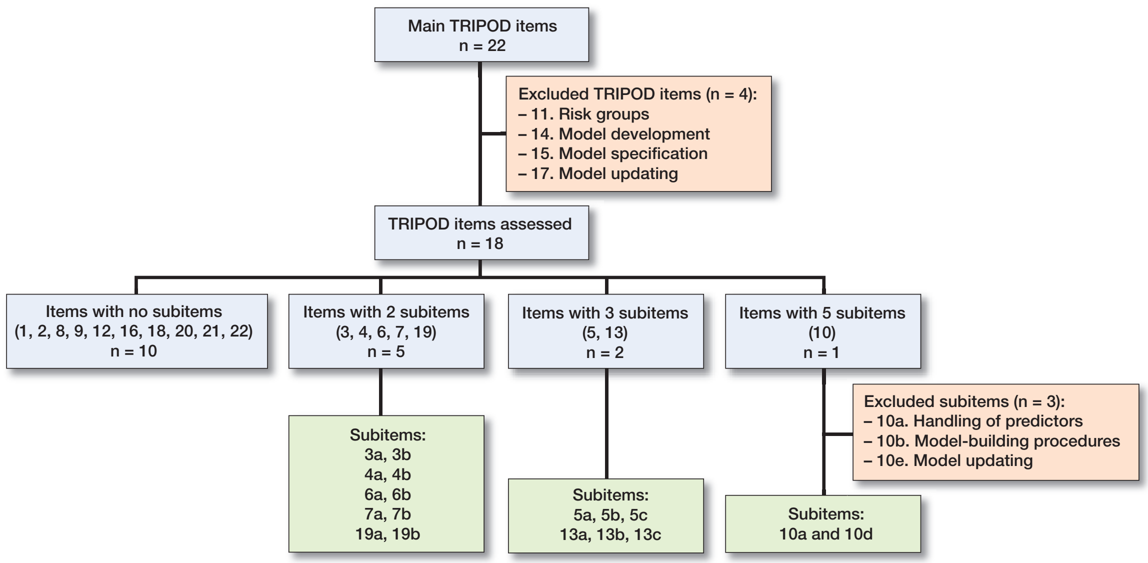

Figure 2 The TRIPOD guidelines list 22 items that are considered essential for informative reporting of prediction models. We used 18 of the 22 main items, because item 11, "Risk groups," was not applicable to any of the external validation studies; item 14 "Model development" and item 15 "Model specification" were not relevant to this review; and item 17 "Model updating" was done in only 1 study. Certain main items consisted of multiple subitems; main items 1, 2, 8, 9, 12, 16, 18, 20, 21, and 22 consisted of no subitems; main items 3, 4, 6, 7, and 19 consisted of 2 subitems (denoted by letters "a" and "b"; e.g., 3a and 3b), main items 5 and 13 consisted of 3 subitems (e.g., 5a, 5b, and 5c), and main item 10 consisted of 5 subitems. However, the subitems 10a "handling of predictors," 10b "model-building procedures," and 10e "model-updating" were also not rated as they were not relevant to this review. Main items and subitems are under the same nomenclature "items" in the manuscript. In total, 28 items could be rated. Overall TRIPOD completeness was calculated per study and each separate item.

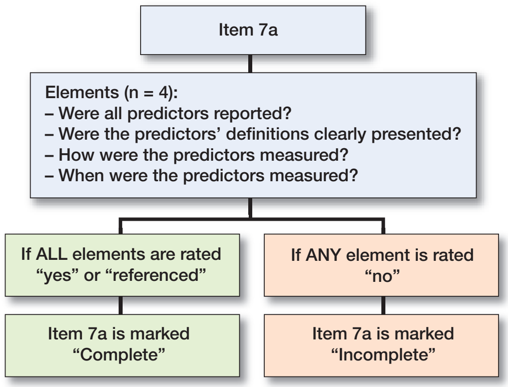

Figure 3 Each item may consist of multiple elements. Each element was rated as "yes," "no," "referenced," or "not applicable." For an item to be considered incomplete, only 1 of the elements needed to be rated as "no." For an item to be considered complete, all of the elements needed to be rated as "yes," "referenced," or at least 1 of the previous 2 with the others "not applicable." For example, item 7a "defining predictors" consisted of 4 elements; (1) were all predictors reported; (2) were the predictors' definitions clearly presented; (3) how were the predictors measured; and (4) when were the predictors measured. Item 7a was considered incomplete if only 1 of the 4 elements were rated "no."

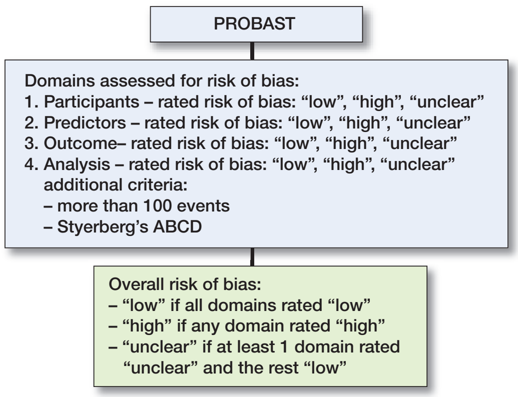

Figure 4. 4 domains are assessed for risk of bias: (1) participants; (2) predictors; (3) outcome; (4) and analysis. Each domain has several signaling questions to guide the rater towards a judgement. The 4 domains are rated as "low," "high," or "unclear" risk of bias. "Unclear" indicates that the reported information is insufficient—no reliable judgement on low or high risk of bias can be made with the information provided. To adapt the PROBAST specifically to our study purposes, we assigned a high risk of bias for the analysis domain (1) if the sample size was too small for the suggested minimum of 100 events in each outcome group (Vergouwe et al. 2005), or (2) when performance measures were not assessed according to Steyerberg's structured stepwise ABCD approach (Steyerberg and Vergouwe 2014). The number of 100 events in each group was deemed essential for the reliable evaluation of calibration plots. The validity of a prediction model was ideally assessed by 4 key metrics to evaluate the performance: calibration slope and intercept (or calibration curve), discrimination with an AUC, and clinical usefulness, with decision-curve analysis. The ratings of all 4 domains resulted in an overall judgement regarding risk of bias. Low overall risk of bias was assigned if each domain scored low. High overall risk of bias was assigned if at least 1 domain was judged to be at high risk of bias. Unclear overall risk of bias was noted if at least 1 domain was judged unclear and all other domains were low. The risk of bias for the 4 domains and overall judgement were reported, not the signaling questions.

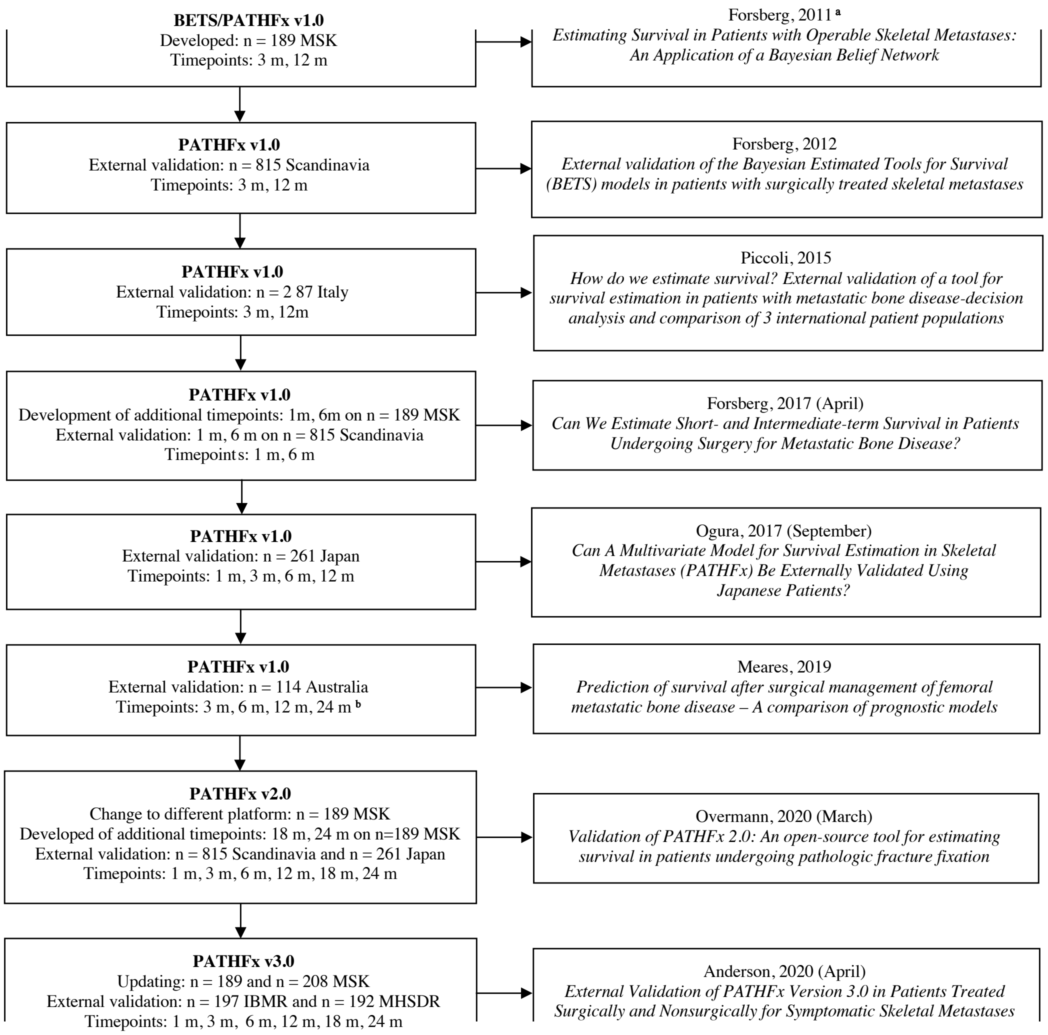

Figure 5. Flowchart of all PATHFx studies for development, external validation, and updating (n = 7). [a] This study is only development and not included in this systematic review. [b] It is unclear which PATHFx model for timepoint 24 m was taken, as the 24 m outcome PATHFx model was developed a year later by Overmann et al. in 2020. MSK = Memorial Sloan Kettering; m = month(s); IBMR = International Bone Metastasis Registry; MHSDR = Military Health System Data Repository.

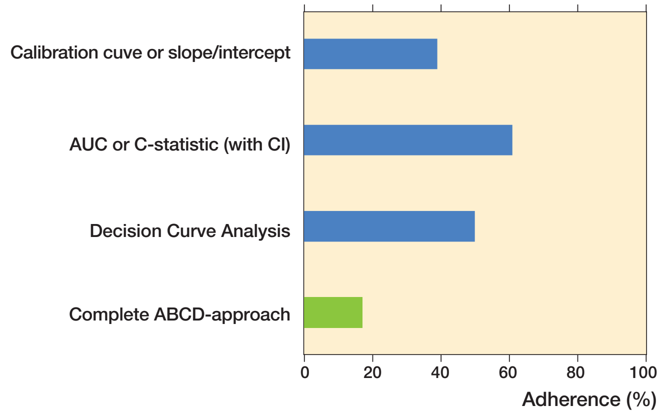

Figure 7. Performance measure of external validation studies according to the ABCD rule and the Brier score. All provided AUC were ROC-AUC.

Table 3. Performance measure of external validation studies compared with developmental studies according to the ABCD approach

Model/ institution	Outcome	Time-points	First author, year	AUC	Calibration intercept, slope	Decision curve analysis	First author, year	AUC	Calibration intercept, slope	Decision curve analysis	Model up-dating
Cleveland	LOS, discharge disposition	nr	Ramkumar, 2019a	0.80<br>0.70	na	na	Same	0.82<br>0.79	na	na	no
Cleveland	LOS; discharge disposition	nr	Ramkumar, 2019b	0.83<br>0.69	na	na	Same	0.75<br>0.76	na	na	no
BETS/ PATHFx 1.0	Survival	3 m<br>12 m	Forsberg, 2012	0.79<br>0.76	na	na	Forsberg, 2011	0.85<br>0.83	na	na	no
PATHFx 1.0	Survival	3 m<br>12 m	Piccioli, 2015	0.80<br>0.77	na	yes	Forsberg, 2011	0.85<br>0.83	na	na	no
PATHFx 1.0	Survival	1 m<br>6 m	Forsberg, 2017	0.76<br>0.76	na	yes	Same	na	na	na	no
PATHFx 1.0	Survival	1 m<br>3 m<br>6 m<br>12 m	Ogura, 2017	0.77<br>0.80<br>0.83<br>0.80	na	yes	Forsberg, 2011/ 2017	0.76<br>0.85<br>0.76<br>0.83	na	yes for 1 m and 6 m	no
PATHFx 1.0	Survival	3 m<br>6 m<br>12 m<br>24 m [a]	Meares, 2019	0.70<br>0.70<br>0.71<br>0.75	na	na	Forsberg, 2011/ 2017	0.85<br>0.76<br>0.83<br>na	na	yes for 6 m	
PATHFx 2.0	Survival	1 m<br>3 m<br>6 m<br>12 m<br>18 m<br>24 m [a]	Overmann, 2020	0.78<br>0.79<br>0.78<br>0.80<br>0.80<br>0.82	na	yes	Same	na	curves	na	no [b]
PATHFx 3.0	Survival	1 m<br>3 m<br>6 m<br>12 m<br>18 m<br>24 m [a]	Anderson, 2020	0.70<br>0.77<br>0.77<br>0.78<br>0.79<br>0.82	na	yes	Same	na	curves	na	yes
SafeTKA	Transfusions	2 w	Jo, 2020	0.88	na	na	Same	0.84	na	na	no
SafeTKA	Acute kidney injury	1 w	Ko, 2020	0.89	curves	na	Same	0.78	curves	na	no
SORG	Survival	5 y	Bongers, 2019	0.87	0.97, -0.58	na	Thio, 2018	0.87	1.03, 0.001	na	no
SORG	Survival	5 y	Bongers, 2020a	0.86	0.68, 0.82	yes					
SORG	Survival	3 m<br>12 m	Karhade, 2020	0.75<br>0.77	0.64, -0.10<br>0.77, 0.43	yes	Karhade, 2020	0.83<br>0.89	0.92, 0.04<br>1.26, 0.07	yes	no
SORG	Survival	3 m<br>12 m	Bongers, 2020b	0.81<br>0.84	0.64, -0.07<br>na [c], 0.57	yes					
SORG	Discharge disposition	nr	Stopa, 2019	0.89	1.09, -0.08	yes	Karhade, 2018	0.82	0.94, 0.03	yes	no
Stanford	Survival, cardiac and renal compli-cations	30 d	Harris, 2019	0.69,<br>0.72,<br>0.60	na	na	Same	0.73,<br>0.73,<br>0.78	curves,<br>curves,<br>na	na	no
Zhengzhou	Survival: overall and cancer specific	3 y	Huang, 2019	0.78<br>0.87	curves<br>na	na	Same	0.79<br>0.82	curves,<br>na	na	no

AUC = area under the curve; na = not available; nr = not relevant; d = day(s); w = week(s); m = month(s); y = years;
BETS = Bayesian Estimated Tools for Survival; SORG = Spinal Oncology Research Group.
The performance measures were extracted from the largest and best performing model from the external validation cohort and the corresponding developmental model.
If no developmental performance measures were available for a certain timepoint (e.g., timepoint 1 m in PATHfx), we used the performance measures of the first external validation as "developmental results" for future comparisons.
[a] No study reported on the development of the 24 m survival endpoint.
[b] No updating of the model was performed to our knowledge, but only transitioning to PATHFx 2.0 using open-source software.
[c] 2 different values were provided in the study.

Table 4. Completeness of reporting of individual TRIPOD items, sorted in descending order (n = 18)

TRIPOD item	TRIPOD description	n
Title and abstract		
1	Identify the study as validating a multivariable prediction model, the target population, and the outcome to be predicted	7
2	Provide a summary of objectives, study design, setting, participants, sample size, predictors, outcome, statistical analysis, results, and conclusions	0
Introduction		
3a	Explain the medical context (including whether diagnostic or prognostic) and rationale for validating the multivariable prediction model, including references to existing models	18
3b	Specify the objectives, including whether the study describes the validation of the model	16
Methods		
4a	Describe the study design or source of data (e.g., randomized trial, cohort, or registry data), separately for the validation data set	18
4b	Specify the key study dates, including start of accrual; end of accrual; and, if applicable, end of follow-up	13
5a	Specify key elements of the study setting (e.g., primary care, secondary care, general population) including number and location of centers	13
5b	Describe eligibility criteria for participants	10
5c	Give details of treatments received, if relevant	4
6a	Clearly define the outcome that is predicted by the prediction model, including how and when assessed	10
6b	Report any actions to blind assessment of the outcome to be predicted	17
7a	Clearly define all predictors used in validating the multivariable prediction model, including how and when they were measured	2
7b	Report any actions to blind assessment of predictors for the outcome and other predictors	0
8	Explain how the study size was arrived at	10
9	Describe how missing data were handled (e.g., complete-case analysis, single imputation, multiple imputation) with details of any imputation method	11
10c	Describe how the predictions were calculated	14
10d	Specify all measures used to assess model performance and, if relevant, to compare multiple models (methods)	8
12	Identify any differences from the development data in setting, eligibility criteria, outcome and predictors	8
Results		
13a	Describe the flow of participants through the study, including the number of participants with and without the outcome and, if applicable, a summary of the follow-up time. A diagram may be helpful	4
13b	Describe the characteristics of the participants (basic demographics, clinical features, available predictors), including the number of participants with missing data for predictors and outcome	11
13c	Show a comparison with the development data of the distribution of important variables (demographics, predictors and outcome)	11
16	Report performance measures (with confidence intervals) for the prediction model (results)	4
Discussion		
18	Discuss any limitations of the study (such as nonrepresentative sample, few events per predictor, missing data)	17
19a	Discuss the results with reference to performance in the development data, and any other validation data	17
19b	Give an overall interpretation of the results considering objectives, limitations, results from similar studies and other relevant evidence	18
20	Discuss the potential clinical use of the model and implications for future research	16
Other information		
21	Provide information about the availability of supplementary resources, such as study protocol, web calculator, and data sets	16
22	Give the source of funding and the role of the funders for the present study	18

TRIPOD = Transparent Reporting of a multivariable prediction model for Individual Prognosis Or Diagnosis. The complete checklist can be found at https://www.tripod-statement.org/wp-content/uploads/2020/03/TRIPOD-Adherence-assessment-form_V-2018_12.pdf

Appendix. Search syntaxes for the PubMed, Embase, and Cochrane Databases

Pubmed: 3 terms—November 17, 2020—724 hits

((“Foot”[Mesh] OR “Ankle”[Mesh] OR “Knee Joint”[Mesh] OR “Knee”[Mesh] OR “Ankle Joint”[Mesh] OR “Hip”[Mesh] OR “Hip Joint”[Mesh] OR “Hip Prosthesis”[Mesh] OR “Hip Fractures”[Mesh] OR “Shoulder Joint”[Mesh] OR “Shoulder”[Mesh] OR “Shoulder Fractures”[Mesh] OR “Shoulder Dislocation”[Mesh] OR “Elbow”[Mesh] OR “Elbow Joint”[Mesh] OR “Wrist Joint”[Mesh] OR “Spine”[Mesh] OR “Intervertebral Disc Degeneration”[Mesh] OR “Bone Neoplasms”[Mesh] OR “Arthroplasty”[Mesh] OR “Fractures, Bone”[Mesh] OR “Orthopedics”[Mesh] OR “Foot”[Tiab] OR “Ankle”[Tiab] OR Knee[Tiab] OR Hip[Tiab] OR “Shoulder”[Tiab] OR Elbow[Tiab] OR Wrist[Tiab] OR Spina*[Tiab] OR Spine*[tiab] OR “degenerative disc”[Tiab] OR “Bone Neoplasms”[Tiab] OR Arthroplast*[Tiab] OR Fractur*[Tiab] OR Orthop*[Tiab])) AND (“Artificial Intelligence”[Mesh] OR “Machine Learning”[Mesh] OR “Supervised Machine Learning”[Mesh] OR “Neural Networks Computer”[Mesh] OR “Deep Learning”[Mesh] OR “support vector machine”[MeSH Terms] OR “support vector machine”[All Fields] OR “Support Vector Machine”[Mesh] OR naive bayes[tiab] OR “bayesian learning”[tiab] OR neural network*[tiab] OR “support vector”[tiab] OR support vectors[tiab] OR random forest[tiab] OR “deep learning”[tiab] OR “machine prediction”[tiab] OR “machine intelligence”[tiab] OR “computational intelligence”[tiab] OR “computational learning”[tiab] OR “computer reasoning”[tiab] OR “machine learning”[tiab] OR convolutional network*[tiab] OR “artificial intelligence”[tiab]) AND (external validation[Title/Abstract] OR validation[Title/Abstract] OR implemental[Title/Abstract] OR implement[Title/Abstract] OR added[Title/Abstract] OR updating[Title/Abstract])

Embase: 3 terms—November 17, 2020—705 hits

(‘foot’/exp/mj OR ‘ankle’/exp/mj OR ‘knee’/exp/mj OR ‘hip’/exp/mj OR ‘hip prosthesis’/exp/mj OR ‘hip fracture’/exp/mj OR ‘shoulder’/exp/mj OR ‘shoulder fracture’/exp/mj OR ‘shoulder dislocation’/exp/mj OR ‘elbow’/exp/mj OR ‘wrist’/exp/mj OR ‘spine’/exp/mj OR ‘intervertebral disk disease’/exp/mj OR ‘bone tumor’/exp/mj OR ‘arthroplasty’/exp/mj OR ‘fracture’/exp/mj OR ‘orthopedic surgery’/exp/mj OR foot:ab,ti OR ankle:ab,ti OR knee:ab,ti OR hip:ab,ti OR shoulder:ab,ti OR spine:ab,ti OR ‘degenerative disc’:ab,ti OR elbow:ab,ti OR wrist:ab,ti OR ‘bone tumor’:ab,ti OR arthroplasty:ab,ti OR fractur:ab,ti OR orthop:ab,ti) AND (‘external validation’:ab,ti OR ‘validation’:ab,ti OR ‘implemental’:ab,ti OR ‘implement’:ab,ti OR ‘added’:ab,ti OR ‘updating’:ab,ti OR ‘updated’:ab,ti) AND (‘artificial intelligence’/exp/mj OR ‘machine learning’/exp/mj OR ‘supervised machine learning’/exp/mj OR ‘artificial neural network’/exp/mj OR ‘deep learning’/exp/mj OR ‘support vector machine’/exp/mj OR ‘bayesian learning’/exp/mj OR ‘neural network’:ab,ti OR ‘naive bayes’:ab,ti OR ‘beyesian learning’:ab,ti OR ‘support vector’:ab,ti OR ‘support vectorts’:ab,ti OR ‘random forest’:ab,ti OR ‘deep learning’:ab,ti OR ‘machine prediction’:ab,ti OR ‘machine intelligence’:ab,ti OR ‘computational intelligence’:ab,ti OR ‘computer learning’:ab,ti OR ‘computer reasoning’:ab,ti OR ‘machine learning’:ab,ti OR ‘convolutional network’:ab,ti OR ‘artificial intelligence’:ab,ti)

Cochrane: 3 terms—November 17, 2020—43 hits

([mh Foot] OR [mh Knee] OR [mh “Knee Joint”] OR [mh “Ankle Joint”] OR [mh Hip] OR [mh “Hip Joint”] OR [mh “Hip Prosthesis”] OR [mh “Hip Fractures”] OR [mh “Shoulder Dislocation”] OR [mh Elbow] OR [mh “Elbow Joint”] OR [mh “Wrist Joint”] OR [mh Spine] OR [mh “Intervertebral Disk Degeneration”] OR [mh “Bone Neoplasms”] OR [mh Arthroplasty] OR [mh “Fractures, Bone”] OR [mh Orthopedics] OR ((Foot OR Ankle OR Knee OR Hip OR Shoulder OR Elbow OR Wrist OR Spine OR Spina* OR “degenerative disk” OR “Bone Neoplasms” OR Arthroplast* OR Fractur* OR Orthop*):ti,ab,kw)) AND ((“external validation” OR “external” OR “validation*” OR “added” OR “implemental” OR “added” OR “updating” OR “updated” OR “implement”):ti,ab,kw) AND (([mh “Artificial Intelligence”] OR [mh “Machine Learning”] OR [mh “Supervised Machine Learning”] OR [mh “Neural Networks (Computer)”] OR [mh “Deep Learning”] OR [mh “Support Vector Machine”] OR ((“naive bayes” OR “bayesian learning” OR “neural network*” OR “support vector” OR “support vectors” OR “random forest” OR “deep learning” OR “machine prediction” OR “machine intelligence” OR “computational intelligence” OR “computational learning” OR “computer reasoning” OR “machine learning” OR “convolutional network*” OR “artificial intelligence”):ti,ab,kw)))

PubMed: Authors —November 17, 2020—2,765 hits

(“Anderson AB”[Author] OR “Forsberg JA”[Author] OR “Arvind V”[Author] OR “Cho SK”[Author] OR “Azimi P”[Author] OR “Mohammadi HR”[Author] OR “Azhari S”[Author] OR “Bevevino AJ”[Author] OR “Chen CY”[Author] OR “Shi HY”[Author] OR “Durand WM”[Author] OR “Daniels AH”[Author] OR “Fatima N”[Author] OR “Shin JH”[Author] OR “Fontana MA”[Author] OR “MacLean CH”[Author] OR “Healey JH”[Author] OR “Gabriel RA”[Author] OR “Vaida F”[Author] OR “Gowd AK”[Author] OR “Liu JN”[Author] OR “Goyal A”[Author] OR “Bydon M”[Author] OR “Han SS”[Author] OR “Ratliff JK”[Author] OR “Harris AHS”[Author] OR “Giori NJ”[Author] OR “Hopkins BS”[Author] OR “Dahdaleh NS”[Author] OR “Huang Z”[Author] OR “Pei F”[Author] OR “Huber M”[Author] OR “Leidl R”[Author] OR “Kalagara S”[Author] OR “Karhade AV”[Author] OR “Schwab JH”[Author] OR “Bedair HS”[Author] OR “Karnuta JM”[Author] OR “Ramkumar PN”[Author] OR “Katakam A”[Author] OR “Khan O”[Author] OR “Fehlings MG”[Author] OR “Kim JS”[Author] OR “Kukar M”[Author] OR “Silvester T”[Author] OR “Kunze KN”[Author] OR “Levine BR”[Author] OR “Lungu E”[Author] OR “Desmeules F”[Author] OR “Martini ML”[Author] OR “Cardi JM”[Author] OR “Merali ZG”[Author] OR “Merrill RK”[Author] OR “Ndu A”[Author] OR “Nwachukwu BU”[Author] OR “Nho SJ”[Author] OR “Ogink PT”[Author] OR “Ottenbacher KJ”[Author] OR “Granger CV”[Author] OR “Paulino Pereira NR”[Author] OR “Pua YH”[Author] OR “Yeo SJ”[Author] OR “Krebs VE”[Author] OR “Scheer JK”[Author] OR “Ames CP”[Author] OR “Schwartz MH”[Author] OR “Verner JJ”[Author] OR “Shi L”[Author] OR “Wang YS”[Author] OR “Siccoli A”[Author] OR “Staartjes VE”[Author] OR “Thio QCBS”[Author] OR “Wu HY”[Author] OR “Ting CK”[Author] OR “Zhang Y”[Author] OR “Hu J”[Author] OR “Zhang Y”[Author] OR “Ratliff JK”[Author]) AND (“Artificial Intelligence”[Mesh] OR “Machine Learning”[Mesh] OR “Supervised Machine Learning”[Mesh] OR “Neural Networks Computer”[Mesh] OR “Deep Learning”[Mesh] OR “support vector machine”[MeSH Terms] OR “support vector machine”[All Fields] OR “Support Vector Machine”[Mesh] OR naive bayes[tiab] OR “bayesian learning”[tiab] OR neural network*[tiab] OR “support vector”[tiab] OR support vectors[tiab] OR random forest[tiab] OR “deep learning”[tiab] OR “machine prediction”[tiab] OR “machine intelligence”[tiab] OR “computational intelligence”[tiab] OR “computational learning”[tiab] OR “computer reasoning”[tiab] OR “machine learning”[tiab] OR convolutional network*[tiab] OR “artificial intelligence”[tiab])

Embase: Authors November 17, 2020—3,235 hits

('Anderson A.B.' OR 'Forsberg J.A.' OR 'Arvind V.' OR 'Cho S.K.' OR 'Azimi P.' OR 'Mohammadi H.R.' OR 'Azhari S.' OR 'Bevevino A.J.' OR 'Chen C.-Y.' OR 'Shi H.-Y.' OR 'Durand W.M.' OR 'Daniels A.H.' OR 'Fatima N.' OR 'Shin J.H.' OR 'Fontana M.A.' OR 'MacLean C.H.' OR 'Healey J.H.' OR 'Gabriel R.A.' OR 'Vaida F.' OR 'Gowd A.K.' OR 'Liu J.N.' OR 'Goyal A.' OR 'Bydon M.' OR 'Han S.S.' OR 'Ratliff J.K.' OR 'Harris A.H.S.' OR 'Giori N.J.' OR 'Hopkins B.S.' OR 'Dahdaleh N.S.' OR 'Huang Z.' OR 'Pei F.' OR 'Huber M.' OR 'Leidl R.' OR 'Kalagara S.' OR 'Karhade A.V.' OR 'Schwab J.H.' OR 'Bedair H.S.' OR 'Karnuta J.M.' OR 'Ramkumar P.N.' OR 'Katakam A.' OR 'Khan O.' OR 'Fehlings M.G.' OR 'Kim J.S.' OR 'Kukar M.' OR 'Silvester T.' OR 'Kunze K.N.' OR 'Levine B.R.' OR 'Lungu E.' OR 'Desmeules F.' OR 'Martini M.L.' OR 'Cardi J.M.' OR 'Merali Z.G.' OR 'Merrill R.K.' OR 'Ndu A.' OR 'Nwachukwu B.U.' OR 'Nho S.J.' OR 'Ogink P.T.' OR 'Ottenbacher K.J.' OR 'Granger C.V.' OR 'Paulino Pereira N.R.' OR 'Pua Y.-H.' OR 'Yeo S.-J.' OR 'Krebs V.E.' OR 'Scheer J.K.' OR 'Ames C.P.' OR 'Schwartz M.H.' OR 'Verner J.J.' OR 'Shi L.' OR 'Wang Y.S.' OR 'Siccoli A.' OR 'Staartjes V.E.' OR 'Thio Q.C.B.S.' OR 'Wu H.-Y.' OR 'Ting C.-K.' OR 'Zhang Y.' OR 'Hu J.' OR 'Zhang Y.' OR 'Ratliff J.K.') AND ('artificial intelligence'/exp/mj OR 'machine learning'/exp/mj OR 'supervised machine learning'/exp/mj OR 'artificial neural network'/exp/mj OR 'deep learning'/exp/mj OR 'support vector machine'/exp/mj OR 'bayesian learning'/exp/mj OR 'neural network':ab,ti OR 'naive bayes':ab,ti OR 'beyesian learning':ab,ti OR 'support vector':ab,ti OR 'support vectorts':ab,ti OR 'random forest':ab,ti OR 'deep learning':ab,ti OR 'machine prediction':ab,ti OR 'machine intelligence':ab,ti OR 'computational intelligence':ab,ti OR 'computer learning':ab,ti OR 'computer reasoning':ab,ti OR 'machine learning':ab,ti OR 'convolutional network':ab,ti OR 'artificial intelligence':ab,ti)

Cochrane: Authors—November 17, 2020—42 hits

((("Ashley B Anderson"):au OR ("AB Anderson"):au OR ("Jonathan A Forsberg"):au OR ("JA Forsberg"):au OR ("Varun Arvind"):au OR ("V Arvind"):au OR ("Samuel K Cho"):au OR ("SK Cho"):au OR ("Parisa Azimi"):au OR ("P Azimi"):au OR ("Hasan R Mohammadi"):au OR ("HR Mohammadi"):au OR ("Shirzad Azhari"):au OR ("S Azhari"):au OR ("Adam J Bevevino"):au OR ("AJ Bevevino"):au OR ("Cheng-Yen Chen"):au OR ("CY Chen"):au OR ("Hon-Yi Shi"):au OR ("HY Shi"):au OR ("Wesley M Durand"):au OR ("WM Durand"):au OR ("Alan H Daniels"):au OR ("AH Daniels"):au OR ("Nida Fatima"):au OR ("N Fatima"):au OR ("John H Shin"):au OR ("JH Shin"):au OR ("Mark A Fontana"):au OR ("MA Fontana"):au OR ("Catherine H MacLean"):au OR ("CH MacLean"):au OR ("John H Healey"):au OR ("JH Healey"):au OR ("Rodney A Gabriel"):au OR ("RA Gabriel"):au OR ("Florin Vaida"):au OR ("F Vaida"):au OR ("Anirudh K Gowd"):au OR ("AK Gowd"):au OR ("Joseph N Liu"):au OR ("JN Liu"):au OR ("Anshit Goyal"):au OR ("A Goyal"):au OR ("Mohamad Bydon"):au OR ("M Bydon"):au OR ("Summer S Han"):au OR ("SS Han"):au OR ("John K Ratliff"):au OR ("JK Ratliff"):au OR ("Alex HS Harris"):au OR ("AHS Harris"):au OR ("Nicholas J Giori"):au OR ("NJ Giori"):au OR ("Benjamin S Hopkins"):au OR ("BS Hopkins"):au OR ("Nader S Dahdaleh"):au OR ("NS Dahdaleh"):au OR ("ZeYu Huang"):au OR ("Z Huang"):au OR ("FuXing Pei"):au OR ("F Pei"):au OR ("Manuel Huber"):au OR ("M Huber"):au OR ("Reiner Leidl"):au OR ("R Leidl"):au OR ("Saisanjana Kalagara"):au OR ("S Kalagara"):au OR ("Aditya V Karhade"):au OR ("AV Karhade"):au OR ("Joseph H Schwab"):au OR ("JH Schwab"):au OR ("Hany S Bedair"):au OR ("HS Bedair"):au OR ("Jaret M Karnuta"):au OR ("JM Karnuta"):au OR ("Prem N Ramkumar"):au OR ("PN Ramkumar"):au OR ("Akhil Katakam"):au OR ("A Katakam"):au OR ("Omar Khan"):au OR ("O Khan"):au OR ("Michael G Fehlings"):au OR ("MG Fehlings"):au OR ("Jun S Kim"):au OR ("JS Kim"):au OR ("Matjaz Kukar"):au OR ("M Kukar"):au OR ("Tomaz Silvester"):au OR ("T Silvester"):au OR ("Kyle N Kunze"):au OR ("KN Kunze"):au OR ("Brett R Levine"):au OR ("BR Levine"):au OR ("Eugen Lungu"):au OR ("E Lungu"):au OR ("Francois Desmeules"):au OR ("F Desmeules"):au OR ("Michael L Martini"):au OR ("ML Martini"):au OR ("John M Cardi"):au OR ("JM Cardi"):au OR ("Zamir G Merali"):au OR ("ZG Merali"):au OR ("Robert K Merrill"):au OR ("RK Merrill"):au OR ("Anthony Ndu"):au OR ("A Ndu"):au OR ("Benedict U Nwachukwu"):au OR ("BU Nwachukwu"):au OR ("Shane J Nho"):au OR ("SJ Nho"):au OR ("Paul T Ogink"):au OR ("PT Ogink"):au OR ("Kenneth J Ottenbacher"):au OR ("KJ Ottenbacher"):au OR ("Carl V Granger"):au OR ("CV Granger"):au OR ("Nuno R Paulino Pereira"):au OR ("NR Paulino Pereira"):au OR ("Yong-Hao Pua"):au OR ("YH Pua"):au OR ("Seng-Jin Yeo"):au OR ("SJ Yeo"):au OR ("Viktor E Krebs"):au OR ("VE Krebs"):au OR ("Justin K Scheer"):au OR ("JK Scheer"):au OR ("Christopher P Ames"):au OR ("CP Ames"):au OR ("Mark H Schwartz"):au OR ("MH Schwartz"):au OR ("James J Verner"):au OR ("JJ Verner"):au OR ("L Shi"):au OR ("L Shi"):au OR ("YS Wang"):au OR ("YS Wang"):au OR ("Alessandro Siccoli"):au OR ("A Siccoli"):au OR ("Victor E Staartjes"):au OR ("VE Staartjes"):au OR ("Quirina CBS Thio"):au OR ("QCBS Thio"):au OR ("Hsin-Yun Wu"):au OR ("HY Wu"):au OR ("Chien-Kun Ting"):au OR ("CK Ting"):au OR ("Yu Zhang"):au OR ("Y Zhang"):au OR ("Jun Hu"):au OR ("J Hu"):au OR ("Yi Zhang"):au OR ("Y Zhang"):au OR ("John K Ratliff"):au OR ("JK Ratliff"):au)) AND (([mh "Artificial Intelligence"] OR [mh "Machine Learning"] OR [mh "Supervised Machine Learning"] OR [mh "Neural Networks (Computer)"] OR [mh "Deep Learning"] OR [mh "Support Vector Machine"] OR (("naive bayes" OR "bayesian learning" OR "neural network*" OR "support vector" OR "support vectors" OR "random forest" OR "deep learning" OR "machine prediction" OR "machine intelligence" OR "computational intelligence" OR "computational learning" OR "computer reasoning" OR "machine learning" OR "convolutional network*" OR "artificial intelligence"):ti,ab,kw)))
